# Supplementary material for: Soybean Oil Regulates the Fatty Acid Synthesis II System of Bacillus amyloliquefaciens LFB112 by Activating Acetyl-CoA Levels
Source: Microorganisms. 2023 Apr 29;11(5):1164. doi: 10.3390/microorganisms11051164 (PMC10222032; doi:10.3390/microorganisms11051164)
Supplement: Supplementary file 1 [file microorganisms-11-01164-s001.zip › microorganisms-2295238-supplementary.pdf]

Supplementary Materials

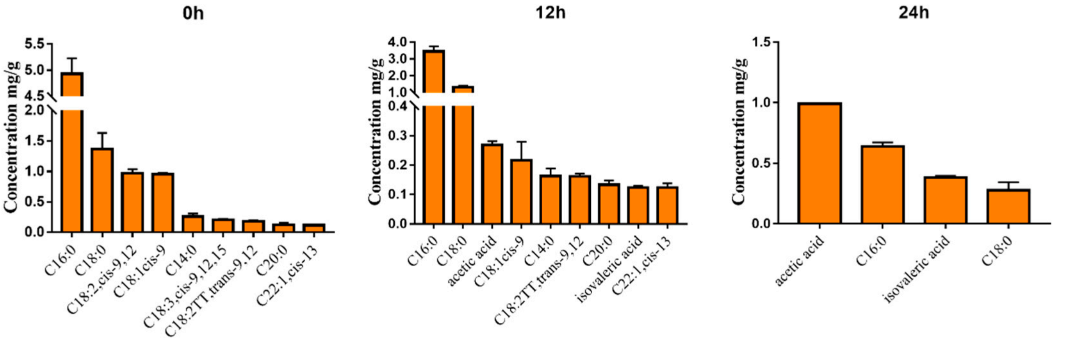

Figure S1. Main types and contents of fatty acids in the supernatant of BPY medium after 0, 12, and 24 h of culture. The content of fatty acid is more than 0.1 mg/mL. Abbreviations of fatty acids:C16:0, Palmitic acid; C18:0, Stearic acid; C18:2, cis-9, 12, Linoleic acid; C18:1cis-9, Oleic acid; C14:0, myristic acid; C18:3, cis-9,12,15, linolenic acid; C18:2 TT, trans -9,12, trans-linoleic acid; C20:0, arachidic acid; C22:1, cis-13, erucic acid.

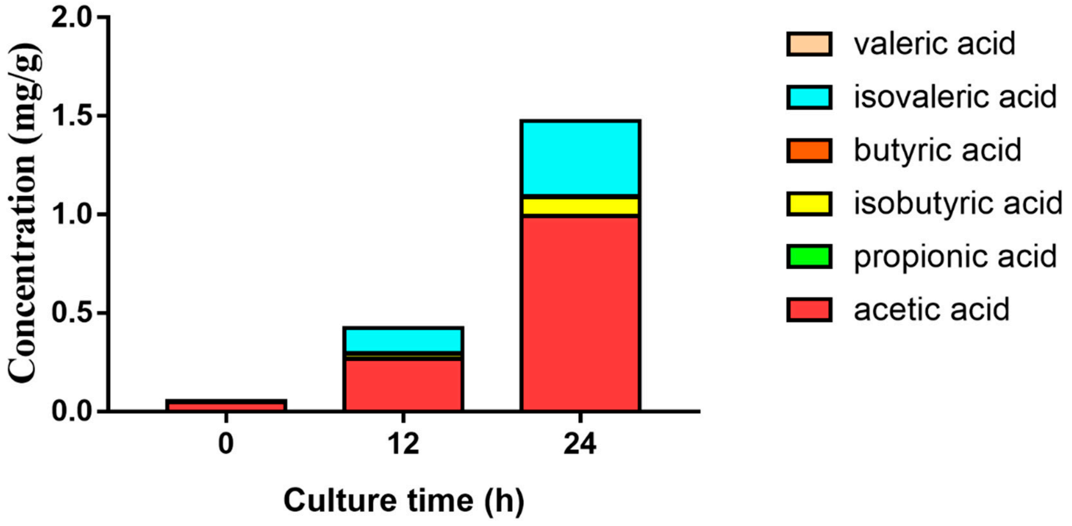

Figure S2. The content of SCFA in the supernatant of BPY medium after 0, 12, and 24 h of culture.

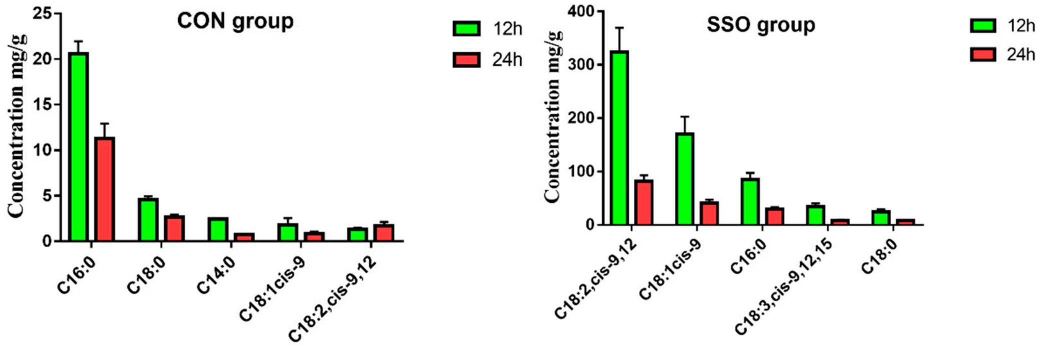

Figure S3. Main types and contents of fatty acids in Bacillus LFB112 pellets after 12, and 24 h of culture. The content of fatty acid is more than 0.1 mg/mL.

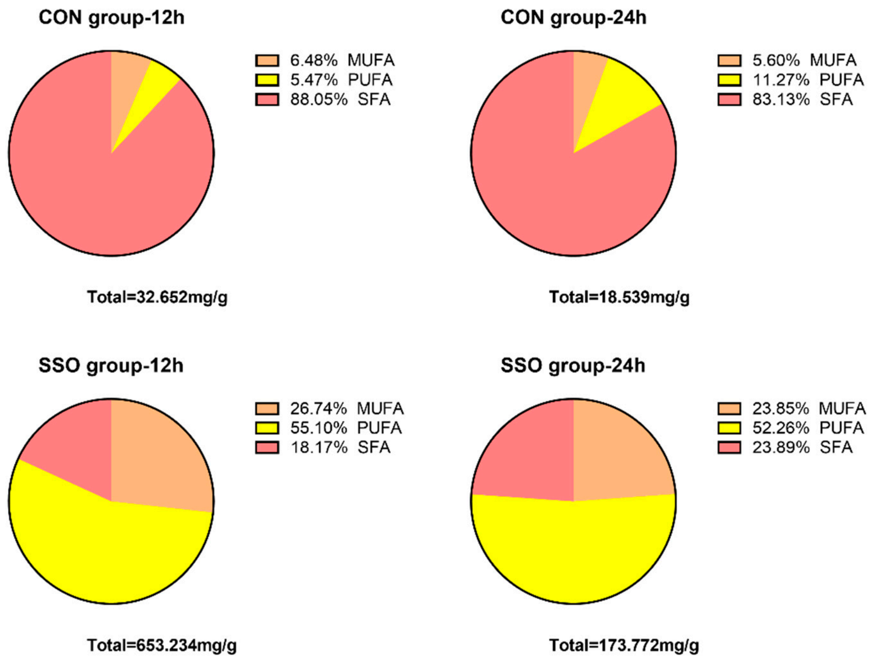

Figure S4. The proportion of fatty acids in Bacillus LFB112 pellets after 0, 12, and 24 h of culture.

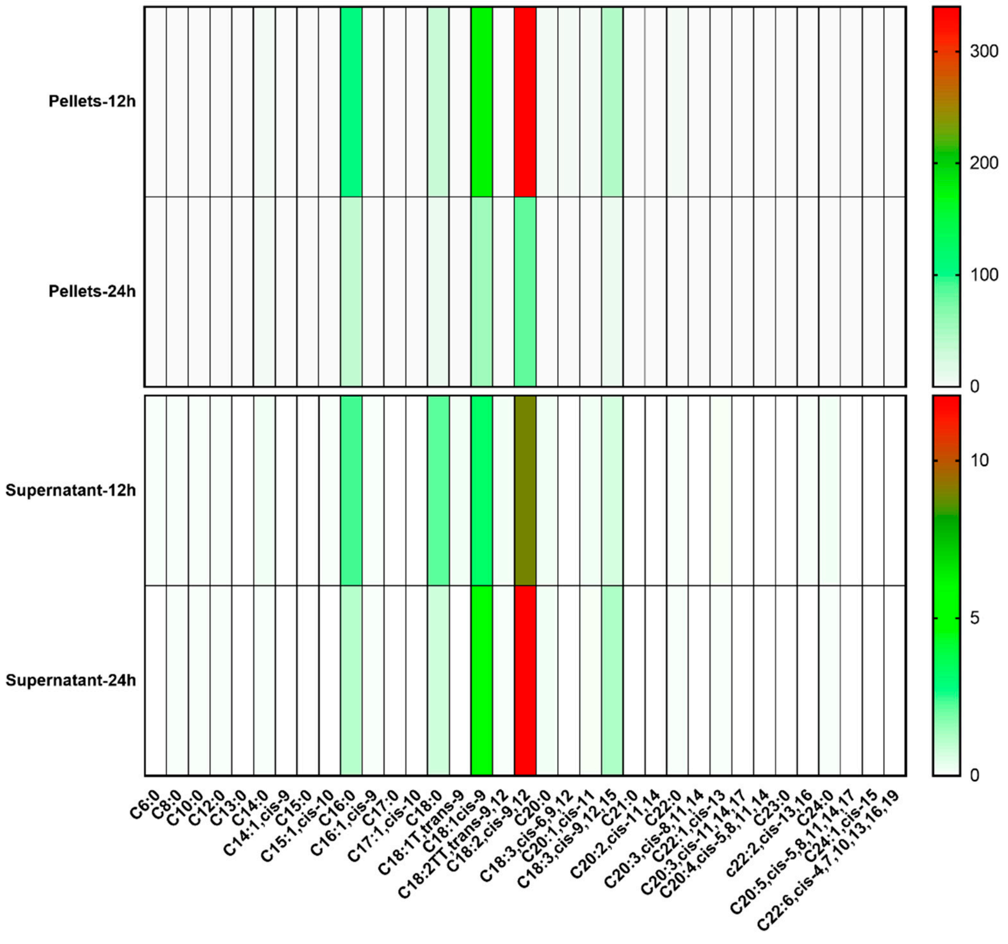

Figure S5. The fatty acid profile between Bacillus LFB112 vitro and vivo.

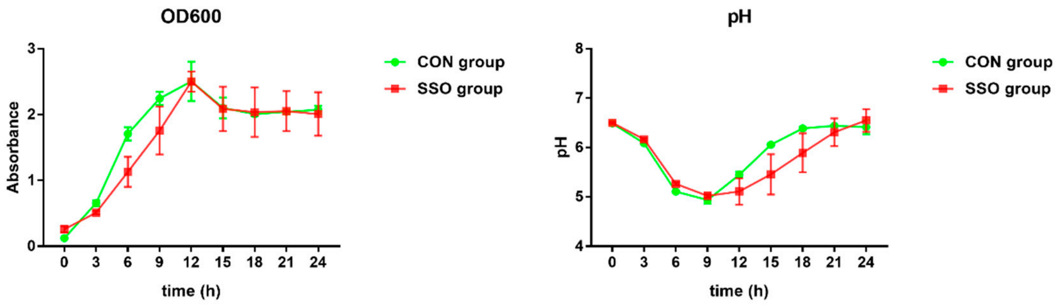

Figure S6. The Growth curve of Bacillus LFB112 under different nutrient conditions.

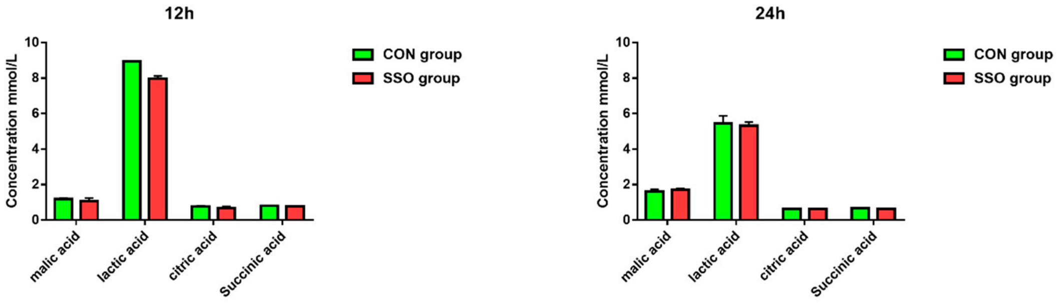

Figure S7. Organic acid content in the TCA cycle.

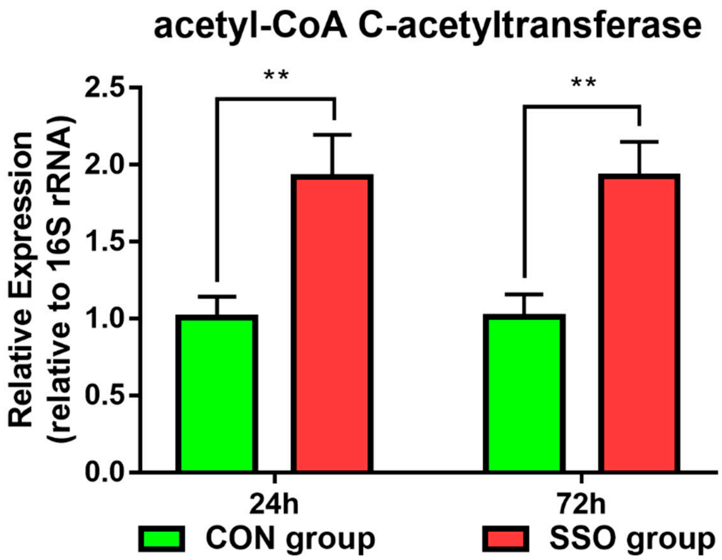

Figure S8. Bacillus LFB112 mRNA levels of *acat-C*. The double asterisk (**) symbol is used to indicate statistical significance at the 0.01 level.

Table S1. Primers used for quantitative real-time PCR

Gene	Accession number	Forward primer (5′ → 3′)	Reverse primer (5′ → 3′)
16S rRNA	FN662486.1	GGCTTCGGCTACCACTTACAGATG	ATCACCCTCTCAGGTCGGCTAC
ack	YP_008951165.1	CGATGCCTGAGCAATCTTACCTGTA	GTATTTATGAGAAGTGCCGTGGAAACC
pyk	YP_008951146.1	CGCCACACGCACGGAAGAAT	CGTCATGCCGACCTGATCTCTG
ldh	YP_008948730.1	CGGTGAAGTAATGGCAAGCGGATT	CTGAATGTCCATGTCGCATAAGTGAGA
acc	YP_008950267.1	TCAATCAGGACCTCTCCTTCATCAGT	GCAGCGGGTACGGTGAAAGAA
fabD	YP_008948648.1	TTACAACTGGAACAATGAGGAATACGC	CTTGACGGCTGACGACCTGATG
fabH	YP_008949391.1	AGAACCGTGCCTGAGGAATGC	CGCTGTGAGGAACGAACCAATC
pptT	YP_008948889.1	TCGACATCGTTGAACTGCACAGA	CGTATTCAGCCTCCGTCAATATTCTT
fabF	YP_008949492.1	GCCCGTTCTTTGTTCCGATGATGA	ATGCCGTCACCGTACAGGAGTT
fabI	YP_008949532.1	GGAAGCATCGTTACACTCACTTACCT	GGCTAAATATCTCACGCTCGCATCA
fabG	YP_008950358.1	ATCATTCGCTGCTGCCGATGTAA	GATGTCAACCTCACCGAGTTCTTCTT
fabZ	YP_008951866.1	TCGCTATCCGTTTCTTCTGGTAGAC	TTTATATCCCGCCGCCCGTTT
acs	YP_008949407.1	CGTTCGGCAGTAATATCGCAAGGT	CGGAACAGATCGCAATTCAGACAGA
acL	YP_008948836.1	CGCCTGAGTTTGACGCTTTATTGAC	GTTGTATATGCCGCCGTGAGTGAT
faaL	YP_008949399.1	TGATTCTCGTCGGCGGATACAATG	CGATGACCACGGCTTCTTTAATGC
acat-C	YP_008951490.1	AAACGGGCAGGCGGCTATGA	ATCCTTGTTCGGCTTCAGGTGTTG

Note: *ackA*: acetate kinase; *pyk*: pyruvate kinase; *ldh*: lactate dehydrogenase; *acc*: acetyl-CoA carboxylase; *fabD*: Malonyl CoA-acyl carrier protein transacylase; *fabH*:3-oxoacyl-ACP synthase III; *pptT*: 4'-phosphopantetheinyl transferase; *fabF*: 3-oxoacyl-ACP synthase II; *fabI*: enoyl-ACP reductase; *fabG*: 3-ketoacyl-ACP reductase; *fabZ*: 3-hydroxyacyl-ACP dehydratase; *acs*: acyl-CoA synthetase; *acL*: acyl-CoA ligase; *faaL*: long-chain fatty acid-CoA ligase. *acat-C*: acetyl-CoA acetyltransferase
